# Ocular biometrics in eyes with different white-to-white corneal diameter in young myopic adults

**DOI:** 10.1038/s41598-024-55318-9

**Published:** 2024-02-27

**Authors:** Li Jiang, Zijing Du, Changting Tang, Shanqing Zhu, Lu Xiong, Xuejun Fang, Jin Zhou, Qingsong Zhang, Wei Sun, Qingyan Zeng, Xiaohua Lei, Zheng Wang, Yijun Hu

**Affiliations:** 1 Refractive Surgery Center, Hankou Aier Eye Hospital, Wuhan, China; 2 Guangdong Eye Institute, Department of Ophthalmology, Guangdong Provincial People’s Hospital (Guangdong Academy of Medical Sciences), Southern Medical University, Guangzhou, China; 3 Aier Institute of Refractive Surgery, Refractive Surgery Center, Guangzhou Aier Eye Hospital, Guangzhou, China; 4 Refractive Surgery Center, Shenyang Aier Eye Hospital, Shenyang, China; 5 Refractive Surgery Center, Chengdu Aier Eye Hospital, Chengdu, China; 6 Refractive Surgery Center, Wuhan Aier Eye Hospital, Wuhan, China; 7 Present Address: Aier Eye Hospital of Wuhan University (Wuhan Aier Eye Hospital), Wuhan, Hubei Province China

**Keywords:** Ocular biometrics, Corneal biometrics, Refractive surgery, White-to-white corneal diameter (WTW), Myopia, Health care, Medical research, Risk factors

## Abstract

The interactions between white-to-white corneal diameter (WTW) and other ocular biometrics are important for planning of refractive surgery and understanding of ocular structural changes in myopia, but such interactions are rarely investigated in young myopic adults. This is a retrospective study involving 7893 young myopic adults from five centers. WTW and other ocular biometrics were measured by Pentacam. The ocular biometrics included anterior corneal curvature (AK) and posterior corneal curvature (PK), central corneal thickness (CCT) and corneal volume (CV), anterior and corneal eccentricity and asphericity, anterior corneal astigmatism (ACA) and posterior corneal astigmatism, anterior chamber depth (ACD), and anterior chamber volume (ACV). The ocular biometrics were compared among eyes of different WTW quartiles. Multivariate linear regression was used to assess the linear associations between WTW and other ocular biometrics adjusting for age, gender and spherical equivalent. In eyes of different WTW quartiles, other ocular biometrics were also significantly different (all *P* < 0.05). After adjusting for age, gender and spherical equivalent, WTW was positively correlated to AK (β = 0.26 to 0.29), ACA (β = 0.13), anterior corneal asphericity (β = 0.05), PK (β = 0.33 to 0.34), posterior corneal asphericity (β = 0.13), ACD (β = 0.29), and ACV (β = 40.69), and was negatively correlated to CCT (β = − 6.83), CV (β = − 0.06 to − 0.78), anterior corneal eccentricity (β = − 0.035), and posterior corneal eccentricity (β = − 0.14) (all *P* < 0.001). In conclusion, we found that in young myopic adults, larger WTW was associated with thinner corneal thickness, flatter corneal curvature, more anterior corneal toricity, less corneal eccentricity and asphericity, and broader anterior chamber. Our findings may fill in the gap of literature, and help us better understand how the anterior segment structures interact with the WTW in myopia.

## Introduction

White-to-white corneal diameter (WTW) is the distance of the horizontal borders of the corneal limbus. WTW is an indicator of the size of the cornea, and is important for investigation of ocular growth^1^. WTW is also essential for evaluation and planning of refractive surgery, such as calculation of intraocular lens (IOL) power^2^ and determination of size of implantable collamer lens (ICL)^3^.

Young myopic adults are the main source of refractive surgery candidates. China has the largest myopic population, and refractive surgery is becoming more and more popular in young myopic adults. WTW has been found to be correlated with corneal tomographic and biomechanical indices^4,5^ which are important for screening of keratoconus before corneal refractive surgery^6^. WTW is also one of the key parameters in planning of ICL implantation^7^, which has gained increased popularity in recent years.

Ocular biometrics may vary in eyes with different WTW. In a previous study, larger WTW was associated with longer corneal curvature radius and longer axial length in children aged 4–18 years^1^. In an older population of 23,627 Chinese cataract patients, WTW was positively correlated with corneal curvature and anterior chamber depth (ACD), and was negatively correlated with lens thickness (LT) and central corneal thickness (CCT)^8^ In eyes with myopia, not only is the axial length elongated, but also is the WTW changed^8,9^. With the alterations of WTW, other ocular biometrics may also be changed^10^. The interactions between WTW and other ocular biometrics are important in understanding the pathogenesis of myopia. However, neither myopic children nor myopic elders are ideal for this purpose, due to unstable refraction or confounding effects of aging. In young myopic adults undergoing refractive surgery, the refraction and ocular biometrics are usually stable, thus this population is better for understanding the interactions between WTW and other ocular biometrics in myopia. However, no previous studies have investigated such interactions in young myopic adults. In the present study, we aimed to investigate ocular biometrics in eyes with different WTW in a large number of young Chinese myopic adults.

## Methods

### Participants

The participants were from a retrospective study described previously^9,11,12^ The study was approved by the Institutional Review Board (IRB) of each center, including Guangzhou Aier Eye Hospital (GZ), Shenyang Aier Eye Hospital (SY), Wuhan Aier Eye Hospital (WH), Chengdu Aier Eye Hospital (CD) and Hankou Aier Eye Hospital (HK), and was conducted according to the tenets of the Declaration of Helsinki^9,11,12^ In brief, young myopic adults undergoing refractive surgery (aged 18–40) were recruited and their medical records were reviewed. The use of contact lenses was discontinued in all cases for screening prior to refractive surgery. Inclusion criteria were myopic adults with a spherical equivalent (SE) ≤ − 0.50 diopter (D) and good quality Pentacam scans. Only the data of the right eyes were included for analysis. This study was only a review of medical records from which patients could not be identified, so the IRBs **(**IRB of GZ, IRB of SY, IRB of WH, IRB of CD, and IRB of HK**) **decided to waive the requirement to get informed consent^9,11,12^. Patients were excluded if they had coexisting corneal diseases, such as keratoconus and forme fruste keratoconus, previous ocular surgery or trauma, severe dry eye, uveitis, glaucoma, wearing soft contact lenses within 2 weeks or rigid gas-permeable lenses within 1 month before examination^9,11,12^.

### Examinations

All patients underwent detailed ophthalmic examinations, including best-corrected visual acuity (BCVA), intraocular pressure (IOP), manifest and cycloplegic refraction, slit-lamp examination of the anterior segment, corneal topography, and Pentacam measurements^9,11,12^.

WTW and other ocular biometrics were measured by and exported from Pentacam (Oculus GmbH, Wetzlar, Germany). The Pentacam device has excellent reproducibility according to previous studies^13,14^. We followed strict quality control procedures to ensure the consistency and reliability of Pentacam measurements across different centers. These procedures included regular calibration and maintenance of the Pentacam devices, training in standardized measurement techniques, data quality checks, and the exclusion of any measurements that did not meet our predefined criteria for reliability^9,15,16^ The following ocular biometrics were included for analysis: WTW, anterior corneal curvature (AK), anterior corneal astigmatism (ACA), CCT, corneal volume (CV) at 3 mm, 5 mm, and 7 mm area, anterior corneal eccentricity and asphericity, posterior corneal curvature (PK), posterior corneal astigmatism (PCA), posterior corneal eccentricity and asphericity, ACD, and anterior chamber volume (ACV).

### Statistical analysis

Distributions of the data were evaluated by Kolmogorov–Smirnov (KS) test. Comparisons of other ocular biometrics among different WTW quartiles were performed by Kruskal–Wallis test. Multiple comparisons were conducted using the Dunn-Bonferroni test. Multivariate linear regression and Bonferroni correction were used to assess the linear associations between WTW and other ocular biometrics adjusting for age, gender and SE, and the regression coefficients (β) were presented. *P* < 0.05 was considered to be statistically significant.

### Ethics statement

This study was conformed to the tenets of the Declaration of Helsinki and was approved by the IRB of Guangzhou Aier Eye Hospital (GZAIER2019IRB20), Shenyang Aier Eye Hospital (2021-001-01), Wuhan Aier Eye Hospital (2019IRBKY05), Chengdu Aier Eye Hospital (IRB20190005) and Hankou Aier Eye Hospital (HKAIER2019IRB-006-01). This study was only a review of medical records from which patients could not be identified, so the IRBs decided to waive the requirement to get informed consent.

## Results

### Demography

**Figure 1:**
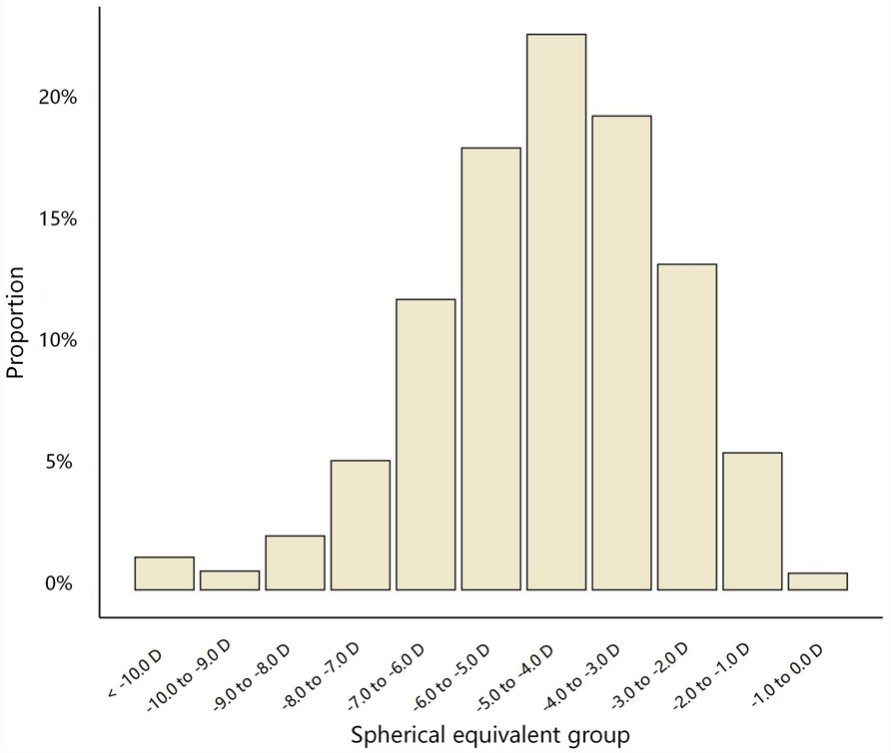
Histogram of spherical error distribution.

**Figure 2:**
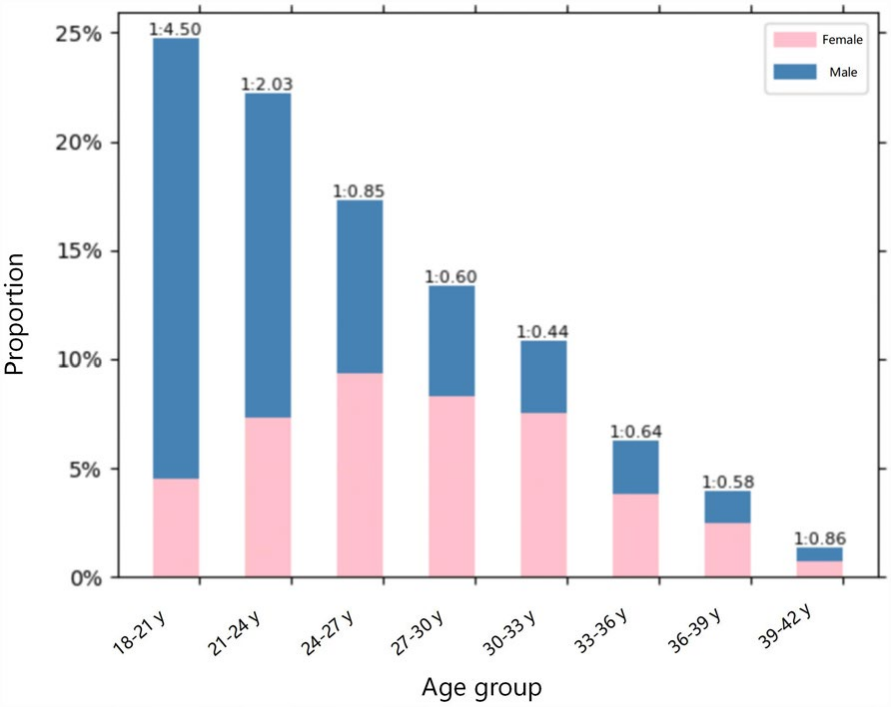
Stacked proportions of males and females by age group. Histogram displaying the stacked proportions of males and females in each age group, with the y-axis representing the proportion of each age group in the total population.

The mean WTW was 11.65 ± 0.38 mm in the study population. Demography of the eyes in different WTW quartiles is shown in Table 1. Mean age and gender distribution were significantly different in the four WTW quartiles, with the oldest age and more females in the first quartile (*P* < 0.001). There were significant differences in SE among different WTW quartiles, with the highest degree of myopia in the first quartile (*P* < 0.001). Figure 1 illustrates a histogram showing the distribution of SE, while Fig. 2 presents stacked bar charts describing the proportions of males and females across different age groups.

**Table 1:** Demography of the eyes in different WTW quartiles.

	1st quartile	2nd quartile	3rd quartile	4th quartile	*P* value
Number of eyes	2358	1614	2245	1676	
Age (years)*	26.43 ± 5.51	25.36 ± 5.43	24.76 ± 5.26	23.60 ± 4.96	< 0.001
Female (no, %)	1363 (57.80)	792 (49.07)	883 (39.33)	439 (26.19)	< 0.001
Male (no, %)	995 (42.20)	822 (50.93)	1362 (60.67)	1237 (73.81)	< 0.001
Spherical error (D)*	− 5.08 ± 2.17	− 4.81 ± 2.00	− 4.61 ± 1.85	− 4.55 ± 1.80	< 0.001
Astigmatism (D)*	− 0.73 ± 0.62	− 0.74 ± 0.64	− 0.75 ± 0.61	− 0.77 ± 0.60	0.019
Spherical equivalent (D)*	− 5.42 ± 2.24	− 5.16 ± 2.09	− 4.95 ± 1.92	− 4.91 ± 1.86	< 0.001

WTW, white-to-white corneal diameter; D, diopter.

*Presented as mean ± standard deviation.

### Ocular biometrics in eyes of different WTW quartiles

The ocular biometrics were significantly different in eyes of different WTW quartiles (Supplementary Table 1). Eyes in the first WTW quartile had the steepest AK and PK, the thickest CCT and the largest CV, and the highest anterior and corneal eccentricity and asphericity, but the smallest ACA and PCA, the shallowest ACD and the smallest ACV (all *P* < 0.05). Specific inter-group multiple comparisons are presented in Supplementary Fig. 1.

### Correlations of the ocular biometrics with WTW

**Figure 3:**
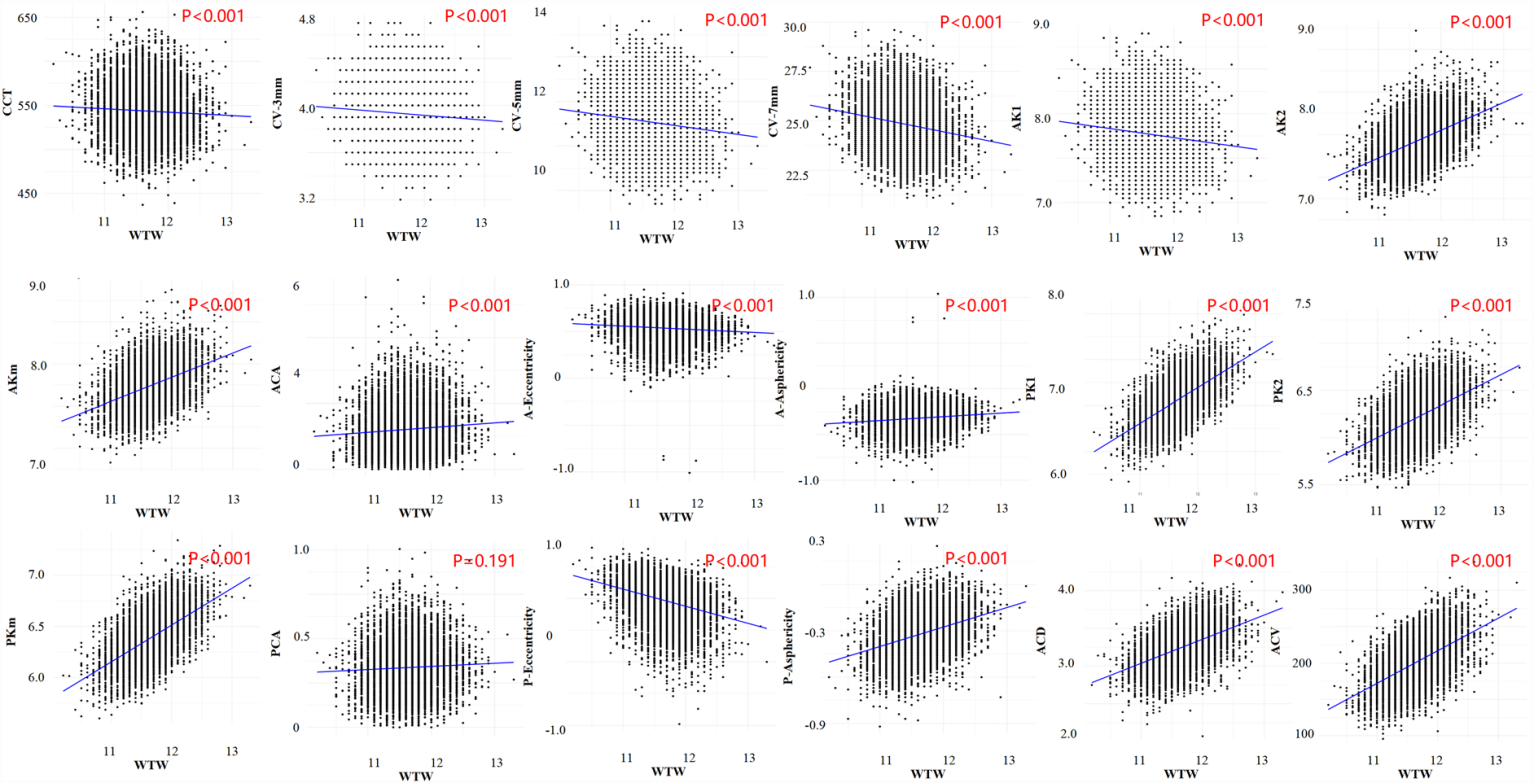
Correlations of the ocular biometrics with WTW. The multivariate linear regression model was adjusted for age, gender, and spherical equivalent. WTW, white-to-white corneal diameter; SD, standard deviation; CCT, central corneal thickness; CV, corneal volume; AK, anterior corneal curvature; ACA, anterior corneal astigmatism; PK, posterior corneal curvature; PCA, posterior corneal astigmatism; ACD, anterior chamber depth; ACV, anterior chamber volume. The *P*-values were after Bonferroni correction and *P* < 0.05 was considered statistically significant.

In univariate linear regression analyses, all of the ocular biometrics were significantly correlated to WTW (all *P-adjusted* < 0.001) (Table 2). After adjusting for age, gender and SE with multivariate linear regression, such correlations were still significant except the PCA (all *P-adjusted* < 0.001) (Table 2 and Fig. 3). Specifically, AK (β = 0.26 to 0.29), ACA (β = 0.13), anterior corneal asphericity (β = 0.05), PK (β = 0.33 to 0.34), posterior corneal asphericity (β = 0.13), ACD (β = 0.29), and ACV (β = 40.69) were positively correlated to WTW. CCT (β = − 6.83), CV (β = − 0.06 to − 0.78), anterior corneal eccentricity (β = − 0.035), and posterior corneal eccentricity (β = − 0.14) were negatively correlated to WTW (Table 2 and Fig. 3).

**Table 2:** Correlations of the ocular biometrics with WTW.

	Univariate linear regression with WTW			Multivariate linear regression with WTW adjusted for age, gender and SE		
	β (95% CI)	R^2^	*P* value*	β (95% CI)	R^2^	*P* value*
Whole cornea						
CCT (μm)	− 3.89 (− 5.55, − 2.22)	0.003	< 0.001	− 6.83 (− 8.54, − 5.12)	0.022	< 0.001
CV-3 mm (mm^3^)	− 0.04 (− 0.05, − 0.03)	0.006	< 0.001	− 0.06 (− 0.08, − 0.05)	0.026	< 0.001
CV-5 mm (mm^3^)	− 0.04 (− 0.05, − 0.03)	0.017	< 0.001	− 0.06 (− 0.08, − 0.05)	0.037	< 0.001
CV-7 mm (mm^3^)	− 0.04 (− 0.05, − 0.03)	0.039	< 0.001	− 0.06 (− 0.08, − 0.05)	0.057	< 0.001
Anterior cornea						
AK1 (mm)	0.33 (0.31, 0.34)	0.248	< 0.001	0.29 (0.28, 0.31)	0.280	< 0.001
AK2 (mm)	0.29 (0.27, 0.30)	0.184	< 0.001	0.26 (0.25, 0.27)	0.210	< 0.001
AKm (mm)	0.31 (0.29, 0.32)	0.225	< 0.001	0.28 (0.26, 0.29)	0.253	< 0.001
ACA (D)	0.15 (0.11, 0.19)	0.007	< 0.001	0.13 (0.09,0.17)	0.048	< 0.001
Eccentricity	− 0.03 (− 0.04, − 0.026)	0.010	< 0.001	− 0.035 (− 0.04, − 0.028)	0.020	< 0.001
Asphericity	0.04 (0.035, 0.05)	0.017	< 0.001	0.05 (0.04, 0.054)	0.029	< 0.001
Posterior cornea						
PK1 (mm)	0.39 (0.38, 0.40)	0.384	< 0.001	0.37 (0.36, 0.38)	0.400	< 0.001
PK2 (mm)	0.33 (0.32, 0.34)	0.266	< 0.001	0.32 (0.31, 0.33)	0.277	< 0.001
PKm (mm)	0.36 (0.35, 0.37)	0.348	< 0.001	0.35 (0.33, 0.36)	0.360	< 0.001
PCA (D)	0.02 (0.01, 0.03)	0.002	< 0.001	0.01 (0.011, 0.024)	0.031	0.191
Eccentricity	− 0.13 (− 0.14, − 0.126)	0.121	< 0.001	− 0.14 (− 0.15, − 0.14)	0.130	< 0.001
Asphericity	0.13 (0.12, 0.14)	0.099	< 0.001	0.13 (0.127, 0.14)	0.108	< 0.001
Anterior chamber						
ACD (μm)	0.32 (0.31, 0.33)	0.223	< 0.001	0.29 (0.27, 0.30)	0.282	< 0.001
ACV (mm^3^)	44.94 (43.38, 46.49)	0.289	< 0.001	40.69 (39.14, 42.24)	0.349	< 0.001

WTW, white-to-white corneal diameter; SE, spherical equivalent; CI, confidential interval; CCT, central corneal thickness; CV, corneal volume; AK, anterior corneal curvature; ACA, anterior corneal astigmatism; PK, posterior corneal curvature; PCA, posterior corneal astigmatism; ACD, anterior chamber depth; ACV, anterior chamber volume.

*P value: The *P*-values after Bonferroni correction. VIF was 1.03–1.20 for the independent variables in multivariate linear regression, suggesting no multicollinearity between the variables.

## Discussion

In the present multi-center study, we showed that ocular biometrics varied in eyes with different WTW, and were linearly correlated to WTW, in a large number of young Chinese myopic adults. Our findings may fill in the gap of literature, which is a lack of study about associations between ocular biometrics and WTW in young adults, since previous studies only involved children or the elderly, in whom the biometrics were either unstable or changed with aging^1,8^

WTW is the horizontal diameter of the cornea, and is also one of the parameters to measure the size of the eyeball. In the present study, the mean WTW was 11.65 ± 0.38 mm, consistent with our previous research^9^ However, it was smaller than measurements reported in Western populations, indicating an ethnic variation in WTW^17,18^ The smaller WTW in our study may be also due to that the participants were myopic. In two previous studies, it was found that WTW was decreased with higher severity of myopia^8,9^ Axial length elongation in myopic patients is associated with changes of the anterior segment structures, such as posterior traction to the limbus, although the underlying mechanisms are unclear^8,9^

Due to the stable refraction and ocular biometrics, young myopic adults undergoing refractive surgery are good candidates to observe the interactions of ocular structures in myopia. At this matter, most previous studies have been about the anterior–posterior elongation of the eye in myopia, but the lateral changes of the eye (such as WTW) are rarely reported. In the present study, we revealed the interactions of WTW with other ocular biometrics in myopia. The findings may help us better understand how other ocular structures (such as the cornea and anterior chamber) are changed when the lateral size of the eye is changed in myopia.

We revealed a negative correlation between CCT and WTW. CCT is an important parameter for screening keratoconus and evaluating safety of corneal refractive surgery^19^ CCT is one of the key parameters in many keratoconus screening or grading systems, such as the latest ABCD classification^20^ However, it seems that none of these systems have considered the correlation between WTW and CCT. According to our findings, the CCT is thinner in eyes with larger WTW. How such correlation influences the accuracy of current diagnosis and grading systems of keratoconus needs to be further investigated.

Previous studies have shown that larger WTW is associated with flatter corneal curvature^1,9^. However, previous studies did not investigate the association of WTW and PK. In the present study, WTW not only was associated with AK, but also was associated with PK. This is may be the first time that the association between WTW and PK is demonstrated. Corneal curvature is also an important parameter in corneal refractive surgery, such as risk evaluation, keratoconus screening and grading^21^ In previous studies, some cut-offs in corneal curvature are used as a diagnostic criteria of keratoconus^22,23^ However, findings from the present study suggest that these cut-offs may need to be adjusted and customized according to the WTW. Further studies are required to investigate the normal range of corneal curvature in eyes of different WTW.

ACA and PCA are the indicators of corneal toricity. In the present study, larger WTW was associated with increased ACA but not PCA, suggesting higher degree of anterior corneal toricity, but not posterior corneal toricity, was associated with larger WTW. However, larger WTW was associated with lower magnitude of the anterior and posterior corneal eccentricity and asphericity. It would be meaningful to investigate the impacts of WTW on outcomes of corneal refractive surgery in future studies.

ACD is associated with WTW, with an β of 0.29 in the present study and 0.16–0.367 in previous studies^8,24,25^ Moreover, WTW is also associated with ACV with an β of 40.69. These findings suggest that the anterior chamber is shallower in eyes with smaller WTW. Anterior chamber biometrics and WTW are important for determining ICL size and evaluating safety of ICL surgery. It has been shown that the Chinese have smaller WTW and shallower anterior chamber than the Western populations^26^ Thus, Chinese myopic patients may have less tolerance of high ICL vault after the surgery, and close monitoring of the anterior chamber biometrics are essential, especially for those with high postoperative vault.

Our findings may have potential clinical implications. Since WTW is correlated to many ocular biometrics (CCT, AK, PK, ACA, ACD and ACV), evaluation of corneal status, screening and risk assessment of keratoconus, and planning of refractive surgery should be performed with the WTW taken into account. For example, cut-offs for some topographic indices to diagnose keratoconus may need to be adjusted according to the WTW. The safety threshold of residual corneal stromal thickness should also be customized according to the WTW.

Our study may have some limitations. First, the study population was limited to young Chinese myopic patients. Whether our findings can be applied to emmetropic eyes and other ethnic groups needs to be confirmed. Second, this is a clinic-based study, and the results need to be validated in population-based studies. Since the examination equipment differs from facility to facility, the current findings also need to be validated in other facilities using different examination equipment. Third, how the associations between WTW and other ocular biometrics influence the outcomes of refractive surgery is unknown and requires further investigations.

In conclusion, we found that larger WTW was associated with thinner corneal thickness, flatter corneal curvature, more anterior corneal toricity, less corneal eccentricity and asphericity, and broader anterior chamber.

### Supplementary Information

Supplementary Information.

## Data Availability

Data are available from the corresponding author upon reasonable request.
