# Supplementary material for: Ocular biometrics in eyes with different white-to-white corneal diameter in young myopic adults
Source: Sci Rep. 2024 Feb 27;14:4720. doi: 10.1038/s41598-024-55318-9 (PMC10899605; doi:10.1038/s41598-024-55318-9)
Supplement: Supplementary file 1 — Supplementary Information. [file 41598_2024_55318_MOESM1_ESM.docx]

**Supplementary Table 1. Ocular biometrics of the eyes in different WTW quartiles**

|  | 1st quartile | 2nd quartile | 3rd quartile | 4th quartile | P value |
| --- | --- | --- | --- | --- | --- |
| **Number of eyes** | 2358 | 1614 | 2245 | 1676 |  |
| **Whole cornea (mean ± SD)** | | | | | |
| CCT (μm) | 545.52 ± 28.40 | 543.75 ± 29.37 | 542.49 ± 28.34 | 542.31 ± 28.11 | <0.001 |
| CV-3mm (mm^3^) | 3.95 ± 0.21 | 3.93 ± 0.21 | 3.92 ± 0.21 | 3.91 ± 0.20 | <0.001 |
| CV-5mm (mm^3^) | 11.62 ± 0.59 | 11.55 ± 0.61 | 11.49 ± 0.59 | 11.44 ± 0.58 | <0.001 |
| CV-7mm (mm^3^) | 25.13 ± 1.24 | 24.91 ± 1.29 | 24.72 ± 1.24 | 24.52 ± 1.22 | <0.001 |
| **Anterior cornea (mean ± SD)** | | | | | |
| AK1 (mm) | 7.80 ± 0.22 | 7.89 ± 0.22 | 7.98 ± 0.21 | 8.12 ± 0.22 | <0.001 |
| AK2 (mm) | 7.61 ± 0.23 | 7.70 ± 0.24 | 7.78 ± 0.24 | 7.90 ± 0.23 | <0.001 |
| AKm (mm) | 7.70 ± 0.22 | 7.80 ± 0.22 | 7.88 ± 0.21 | 8.01 ± 0.22 | <0.001 |
| ACA (D) | 1.15 ± 0.67 | 1.19 ± 0.70 | 1.25 ± 0.68 | 1.28 ± 0.67 | <0.001 |
| Eccentricity | 0.55 ± 0.14 | 0.54 ± 0.14 | 0.54 ± 0.12 | 0.52 ± 0.13 | <0.001 |
| Asphericity | 0.34 ± 0.13 | 0.33 ± 0.13 | 0.32 ± 0.12 | 0.30 ± 0.12 | <0.001 |
| **Posterior cornea (mean ± SD)** | | | | | |
| PK1 (mm) | 6.39 ± 0.20 | 6.51 ± 0.19 | 6.61 ± 0.19 | 6.77 ± 0.19 | <0.001 |
| PK2 (mm) | 6.07 ± 0.21 | 6.17 ± 0.22 | 6.26 ± 0.21 | 6.40 ± 0.22 | <0.001 |
| PKm (mm) | 6.23 ± 0.19 | 6.34 ± 0.19 | 6.44 ± 0.19 | 6.59 ± 0.19 | <0.001 |
| PCA (D) | 0.329 ± 0.14 | 0.337 ± 0.14 | 0.340 ± 0.13 | 0.345 ± 0.13 | 0.002 |
| Eccentricity | 0.54 ± 0.15 | 0.50 ± 0.15 | 0.47 ± 0.16 | 0.41 ± 0.17 | <0.001 |
| Asphericity | -0.38 ± 0.14 | -0.33 ± 0.13 | -0.30 ± 0.13 | -0.25 ± 0.13 | <0.001 |
| **Anterior chamber (mean ± SD)** | |  |  |  |  |
| ACD (μm) | 3.10 ± 0.23 | 3.22 ± 0.22 | 3.29 ± 0.23 | 3.42 ± 0.24 | <0.001 |
| ACV (mm^3^) | 181.82 ± 27.51 | 196.40 ± 25.60 | 207.01 ± 27.06 | 225.73 ± 29.10 | <0.001 |

WTW, white-to-white corneal diameter; SD, standard deviation; CCT, central corneal thickness; CV, corneal volume; AK, anterior corneal curvature; ACA, anterior corneal astigmatism; PK, posterior corneal curvature; PCA, posterior corneal astigmatism; ACD, anterior chamber depth; ACV, anterior chamber volume.


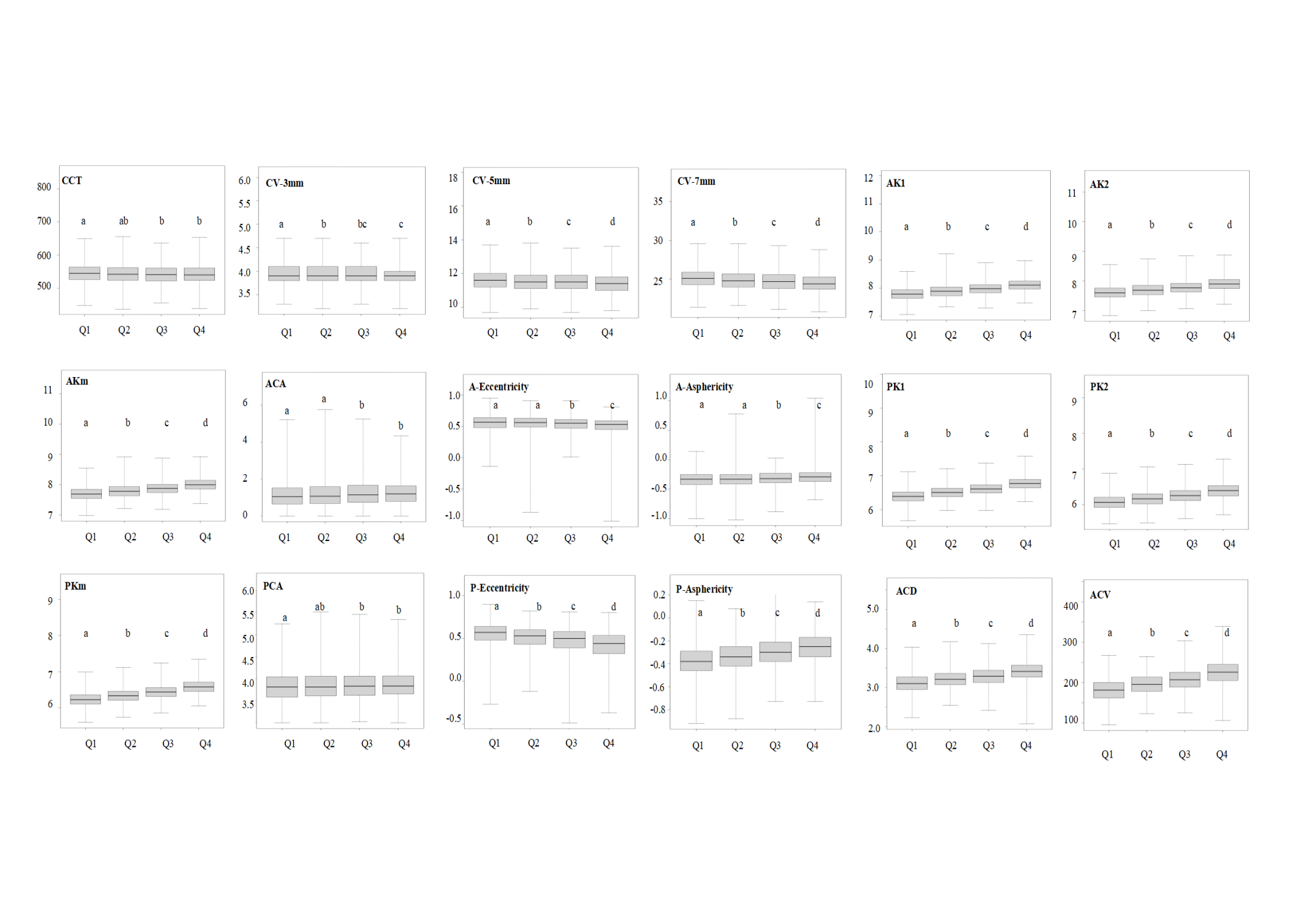


**Supplementary Figure 1.** **Multiple comparisons of Ocular biometrics of the eyes in different WTW quantiles.**

Multiple comparisons were conducted using the Dunn-Bonferroni test. Using letters (e.g., a, ab, b) to represent different levels of significance among groups. The meaning of these letters is as follows: If two groups share the same letter (e.g., a and a), there is no significant difference between them. If two groups have different letters (e.g., a and b), there is a significant difference between them. If two groups share the same letter, but there are other groups with different letters (e.g., ab and a), it means that groups with the same letter do not have significant differences between them, however, there are significant differences between them and groups with different letters. These letters are a way to visually represent the results of post hoc tests and help identify which groups are statistically different from each other.

WTW, white-to-white corneal diameter; SD, standard deviation; CCT, central corneal thickness; CV, corneal volume; AK, anterior corneal curvature; ACA, anterior corneal astigmatism; PK, posterior corneal curvature; PCA, posterior corneal astigmatism; ACD, anterior chamber depth; ACV, anterior chamber volume.
